# 

**Published:** 1874-04-01

**Authors:** 


ILLINOIS STATE MEDICAL SOCIETY.—The next annual meeting of this Society is to be held in Chicago, on Tuesday, the 19th of May next. The profession in the city will cordially welcome their professional brethren from all parts of the State; and we hope to see a much larger number present than at any former meeting since the Society was organized. Let none forget the time, but come, each one prepared to contribute something to the interest of the occasion.

### *BOOKS RECEIVED.*

Treatment of Nervous and Rheumatic Affec, tions by Static Electricity. By Dr. A. Arthius, translated from the French by Dr. J-H. Etheridge, M.D. Chicago: W. B. Keen. Cooke & Co. 144 pp. Price, $2.00.

A Treatise on Therapeutics, comprising Materia Medica and Toxicology. By H. C. Wood, Jr., M.D. Philadelphia: J. B. Lippincott & Co.

Relations of Colorado to Pulmonary Consumption. By Thos. E. Massey, A.M., M.D., Denver, Col. Pamphlet; 26 pp.

